# Intentional Toxic Ingestion of Sodium Fluoride: A Case Report

**DOI:** 10.7759/cureus.5025

**Published:** 2019-06-28

**Authors:** Rachel E Bridwell, Brandon M Carius, Eric B Tomich, Joseph K Maddry

**Affiliations:** 1 Emergency Medicine, Brooke Army Medical Center, Fort Sam Houston, USA; 2 Emergency Services, Highline Medical Center, Burien, USA

**Keywords:** sodium fluoride, toxic ingestion, gastrointestinal hemorrhage, dysrhythmia

## Abstract

Sodium fluoride is an accessible and frequently used compound that if ingested can cause ventricular dysrhythmias, hemorrhage, and death. We present a case report of a 21-year-old female who presented following an intentional ingestion of a lethal dose of sodium fluoride, developing massive hemorrhage and cardiac arrest.

## Introduction

Sodium fluoride (NaF) is a colorless, odorless powder readily available online at 98% pure reagent for use as an insecticide, homemade toothpaste, and wheel cleaner, with an estimated lethal dose of 70-140 mg/kg, or 5-10 g [1-2]. The combination of electrolyte derangements and mucosal corrosion with NaF toxic ingestions (TIs) produces life-threatening complications, including cardiac dysrhythmia, coagulopathy, and gastrointestinal hemorrhage [3-5]. Here, we describe a fatal case of NaF TI.

## Case presentation

A 21-year-old female presented to the ED via emergency medical services (EMS) transport. Her father found the patient unresponsive in a local park, after reportedly consuming a 226 g bottle of 99.9% NaF powder in a suicide attempt. The EMS found the patient in the field with a depressed mental status and intermittent vomiting, and intubated her using 10 mg of etomidate, 100 mg of succinylcholine, 50 mg of rocuronium, and 4 mg of midazolam. 

Upon presentation to the ED, vital signs were a blood pressure of 99/63 mmHg, heart rate of 115 beats per minute (bpm), and pulse oximetry of 100%, with a Glasgow Coma Scale score of 3T. Physical examination was limited due to intubation and sedation, though remaining examination was generally unremarkable. Initial electrocardiogram (ECG) showed sinus tachycardia with a rate of 125 bpm and normal QRS and QTc intervals. Initial laboratory findings showed prominent hypocalcemia of 7.3 mg/dL with ionized calcium of 0.88 mmol/L, and hypomagnesemia of 1.5 mg/dL. Initial hemoglobin (Hgb) and hematocrit (Hct) were 13.6 g/dL and 40%, with a glucose of 140 mg/dL. Additional urine drug screening was negative. An orogastric tube (OGT) was placed with gastric lavage, and 4 g of magnesium and a 1 L bolus of normal saline were given intravenously. Fentanyl and midazolam were used to maintain sedation.

Toxicology consultation recommended large supplemental calcium and magnesium and nephrology consultation. Central vascular access with a Quinton catheter was obtained via the right internal jugular vein. A widening QRS on telemetry prompted additional large doses of calcium and magnesium were administered. Two hours after arrival, the patient lost pulses with ventricular fibrillation. Cardiopulmonary resuscitation (CPR) was initiated, with administration of sodium bicarbonate, magnesium, and calcium. During compressions, the patient maintained good oxygen saturation and demonstrated purposeful motor function. However, repeat rhythm checks revealed asystole or pulseless electrical activity despite multiple doses of epinephrine, prompting a continuous epinephrine infusion.

Large volumes of blood were then noted in the OGT and endotracheal tube. Repeat laboratory findings revealed acute anemia, with Hgb and Hct now 7.1 g/dL and 21%, respectively. Massive transfusion protocol was initiated with four units of packed RBCs immediately given. Despite these interventions, the patient did not achieve return of spontaneous circulation, and resuscitative efforts were terminated 90 min later.

## Discussion

NaF ingestion rapidly generates life-threatening physiologic derangements, including hyperkalemia, hypomagnesemia, and hypocalcemia. While formation of CaF­2 was the proposed mechanism of hypocalcemia, in vitro models showed the main species fluorapatite, Ca5(PO4)3F, consumes five Ca2+ per F-, occurring at a rate that outpaces mobilization of bone [3, 6]. In addition to electrolyte derangements, strong binding affinity to a variety of metalloproteases generates widespread metabolic dysfunction [3]. Aerobic metabolism disruption and resulting metabolic lactic acidosis increase cell membrane permeability, making hyperkalemia more difficult to correct [2-4, 7-9].

Ventricular dysrhythmias occur within 90 min in large ingestions [5, 10-11]. A deadly synthesis of hypocalcemia and hyperkalemia was originally considered the culprit of dysrhythmias [12-14]. However, electrolyte correction does not necessarily reverse cardiotoxicity as multiple cases demonstrate normokalemic ventricular dysrhythmia or normocalcemia with fatal hyperkalemia [3, 8-9, 14-15]. Intrinsic F- toxicity and negative inotropy may instead account for cardiac dysrhythmia [8, 16]. These complex toxicology patients have multiple mechanisms of derangement, requiring frequent electrolyte measurements and ECGs [7].

Hemorrhage is driven in part by prothrombin time prolongation and hypocalcemia coagulopathy. Vascular erosion from F- risks severe hemorrhagic gastritis, necessitating treatment with calcium chloride lavage as part of emergent stabilization [3]. Without control, hemorrhagic emesis can complicate ventilatory and circulatory status [10].

Rapid stabilization is paramount in NaF TIs with precipitous clinical decline. Along with CPR requiring up to 40 defibrillations, early endotracheal intubation facilitates nasogastric tube placement for gastric lavage, hemorrhagic aspirate protection, and enteral calcium administration [3-5, 7, 17]. Lavage goals should target 1 g of calcium using 10% calcium gluconate per gram of calculated fluoride, with frequent cation checks [3]. Case reports demonstrated survival with massive enteral and parenteral calcium administration during resuscitation [18-19]. Other more invasive treatments include endoscopy within 96 h and early hemodialysis for management of refractory hyperkalemia [7, 15].

## Conclusions

In this case, intentional NaF ingestion resulted in rapid clinical deterioration and fatal cardiac dysrhythmia. Early administration of calcium and magnesium, treatment of dysrhythmias, and emergent dialysis for correction of electrolyte imbalances may prevent death.
